# Barriers to the Utilization of mHealth Applications in Saudi Arabia: Insights from Patients with Chronic Diseases

**DOI:** 10.3390/healthcare13060665

**Published:** 2025-03-18

**Authors:** Haitham Alzghaibi

**Affiliations:** Department of Health Informatics, College of Applied Medical Sciences, Qassim University, Buraydah 52571, Saudi Arabia; halzghaibi@qu.edu.sa

**Keywords:** mHealth, patients’ perspectives, digital health, Saudi Arabia, chronic diseases

## Abstract

Background: Mobile health (mHealth) applications play a crucial role in enhancing healthcare accessibility, patient engagement, and chronic disease management. However, technical, usability, accessibility, and privacy-related barriers continue to hinder their widespread adoption. The Sehaty app, a government-managed mHealth platform in Saudi Arabia, is widely used for scheduling medical appointments, accessing health records, and communicating with healthcare providers. Understanding the challenges associated with its utilization is essential for optimizing its functionality and improving user experience. Aim: This study aims to identify and evaluate the key barriers affecting the adoption and usability of the Sehaty mHealth application among patients with chronic conditions in Saudi Arabia. Specifically, it examines challenges related to technical performance, usability, accessibility, privacy, and security and their impact on user satisfaction and engagement. Methods: A cross-sectional study was conducted using a structured questionnaire distributed to 344 participants selected through purposive sampling to ensure the inclusion of active Sehaty users with chronic conditions. The questionnaire assessed 10 primary usability barriers, including technical issues, navigation difficulties, privacy concerns, and accessibility limitations. Descriptive statistics and correlation analyses were performed to evaluate the prevalence and interrelationships of these barriers. Results: The findings indicate that technical barriers, including frequent application crashes, slow responsiveness, and system instability, significantly hinder user satisfaction. Usability challenges, such as difficulties in navigation and task completion, further impede engagement. Moreover, privacy and security concerns emerged as significant deterrents, with users expressing apprehensions about data safety and transparency. Accessibility barriers, particularly for older adults and individuals with disabilities, were associated with insufficient support and training, making the app less user-friendly for these populations. The study highlights the interconnected nature of usability challenges, suggesting that improvements in technical stability and interface design could lead to enhanced user confidence, engagement, and overall satisfaction. Conclusions: Addressing these barriers requires targeted technical enhancements, user-centered design improvements, and strengthened data security measures to promote trust and engagement. Additionally, implementing comprehensive user support systems and accessibility features is essential to ensuring equitable access to mHealth services. While the study’s generalizability is limited by its focus on a single government-managed platform, its findings offer valuable insights applicable to broader mHealth initiatives. Future research should incorporate longitudinal studies to assess the long-term impact of usability improvements on mHealth adoption and healthcare outcomes.

## 1. Introduction

Mobile health (mHealth) applications have emerged as transformative tools in healthcare, offering enhanced access to medical information, real-time monitoring, and seamless communication with healthcare providers [1,2,3,4,5]. Despite their potential, the adoption and sustained use of mHealth apps face substantial challenges spanning technical, usability, financial, and psychological domains. These barriers limit the effectiveness and accessibility of these technologies, particularly among diverse patient populations [5,6,7].

A significant barrier to mHealth adoption is usability. Many patients struggle with complex interfaces and insufficient design considerations, particularly elderly users who may have limited technological literacy, visual impairments, or dexterity issues [4,8]. The time commitment required to learn and navigate these applications further discourages engagement, especially among individuals with busy schedules or low motivation [8].

Digital literacy is another critical factor influencing mHealth adoption. A considerable proportion of users are unaware of these applications or lack the technical skills to use them effectively [8,9]. This digital divide is particularly pronounced among older adults and underserved populations with limited access to technology and digital education [9,10]. Without targeted digital literacy initiatives, these groups remain excluded from the benefits of mHealth solutions.

Privacy and security concerns also deter patients from using mHealth apps. Users often express apprehension regarding unauthorized access to sensitive health data, particularly when dealing with stigmatized conditions such as mental health disorders or HIV/AIDS [1,8]. Many applications lack transparent privacy policies and robust security features, leading to decreased trust among potential users [1].

Another significant challenge is the lack of seamless integration with existing healthcare systems. Many mHealth apps function in isolation, lacking interoperability with electronic health records (EHRs) or healthcare provider systems [8,9]. Patients often have to manually input data, creating additional burdens and reducing engagement [1,11,12].

Financial constraints further impede widespread adoption. While some applications are free, others require subscription fees or in-app purchases that may be unaffordable for certain users [12,13]. Hidden costs within “free” apps, along with the need for high-speed internet and modern mobile devices, add an indirect financial burden, particularly for individuals in low-income settings [4].

The regulatory landscape surrounding mHealth applications remains fragmented and inconsistent across regions. Patients often question accountability in their healthcare management when using these tools, whether responsibility lies with app developers or healthcare providers [1,4]. Ethical concerns related to informed consent, data ownership, and patient autonomy further complicate widespread implementation [1].

Motivation plays a pivotal role in mHealth adoption. Many individuals do not engage consistently with these applications due to a perceived lack of immediate benefits [8]. Time constraints further exacerbate this issue, as busy schedules make it challenging to learn new technologies or regularly input health data [8].

Infrastructure limitations also hinder the effective use of mHealth applications. Rural and remote areas often lack reliable internet connectivity, preventing users from fully utilizing teleconsultations and real-time monitoring features [9]. Poor internet access, outdated mobile devices, and compatibility issues with different operating systems further limit accessibility [1].

### 1.1. The Sehhaty App: A Case Study in Saudi Arabia

The Sehhaty app, developed by the Saudi Ministry of Health (MoH), exemplifies the potential of mHealth applications in transforming healthcare delivery [14]. The Sehaty application (Version 1.3), a government-managed mHealth platform, aligns with this vision by providing users with the ability to schedule medical appointments, access EHRs, monitor test results, and communicate with healthcare professionals remotely. Given its widespread adoption, a comprehensive examination of the usability challenges and adoption barriers associated with Sehaty is crucial to optimizing its effectiveness and ensuring it meets the diverse needs of its users [7,14,15]. Sehhaty played a critical role in Saudi Arabia’s COVID-19 response, facilitating over 24 million testing appointments and administering more than 61 million vaccine doses [16]. Saudi Arabia has prioritized digital health innovations as a key component of its Vision 2030 strategy, which seeks to modernize healthcare services through the integration of advanced digital platforms and telemedicine solutions [16].

With over 24 million users, approximately 68.5% of Saudi Arabia’s population, the Sehhaty app is integral to the country’s digital health transformation aligned with Saudi Vision 2030 [16]. However, barriers to its utilization persist, including usability concerns, digital literacy gaps, and integration challenges. Understanding these obstacles is essential for optimizing the app’s functionality and ensuring equitable healthcare access across the Kingdom [7,17].

### 1.2. Global Perspectives on mHealth Implementation

mHealth has emerged as a transformative tool in healthcare, enhancing patient engagement and improving care delivery. In Europe and the United States, its adoption is supported by strong regulatory frameworks, government initiatives, and advanced digital infrastructure. Policies such as the HITECH Act (United States), DiGA (Germany), and EHDS (Europe) facilitate the integration and reimbursement of digital health tools, fostering widespread implementation [18]. A notable example is the Netherlands’ “The Box” project, which provides cardiac patients with mHealth devices post-discharge, improving remote monitoring and outpatient care. Despite these advancements, challenges remain, including data privacy concerns, interoperability issues, digital literacy gaps, and socioeconomic disparities that hinder equitable access. Addressing these barriers is essential to fully leverage mHealth’s potential in modern healthcare systems [18,19]. Similarly, in Sub-Saharan Africa, mHealth systems have contributed to reducing data collection costs, elderly care expenses, and maternal and perinatal mortality [20].

However, implementation challenges vary across countries. Technical barriers, such as usability issues, system integration failures, and data security concerns, persist. Ensuring user-friendly design and interoperability with existing health systems remains a major challenge. Limited internet connectivity, particularly in rural areas, also constrains the effectiveness of these solutions [1,2,3,5,9,20].

Socioeconomic and cultural factors further influence adoption. Limited access to mobile devices, language barriers, and variations in digital literacy hinder widespread use [20,21]. Cultural attitudes toward technology and healthcare practices also shape acceptance levels [21]. Financial constraints, including the absence of sustainable business models and limited insurance coverage for mHealth services, exacerbate implementation difficulties, particularly in low-income nations [20].

Regulatory and policy inconsistencies present additional challenges. Many countries lack clear digital health regulations, leading to uncertainties in implementation and data governance issues [22,23,24]. The integration of mHealth into national healthcare systems remains a complex process, often requiring parallel reporting structures and specialized workforce training [24].

To address these challenges, several strategies have been proposed. Strengthening government coordination mechanisms, integrating vertical data systems into broader health information frameworks, and increasing transparency in mHealth funding and activities, are crucial. Channeling resources through national institutional frameworks while supporting capacity-building initiatives can further enhance mHealth adoption. Additionally, prioritizing user-centered design is essential to ensure that mHealth interventions meet the diverse needs of target populations [20].

While mHealth presents significant opportunities for improving healthcare accessibility and delivery, its successful implementation requires addressing a complex set of technical, socioeconomic, and systemic barriers. Tailoring solutions to local contexts, fostering multi-sectoral collaborations, and investing in digital health literacy initiatives are critical for maximizing the potential of mHealth. As digital healthcare continues to evolve, ongoing research and policy interventions will be essential in overcoming persistent challenges and ensuring the effective integration of mHealth applications into global healthcare systems.

### 1.3. Aim of Study

To explore the barriers to the implementation of the mHealth application in Saudi Arabia.

What this study adds:

- The research classifies obstacles into 10 factors comprising 45 operationalized items, providing a comprehensive framework for evaluating problems in mobile health applications.
- It underscores the interconnection of hurdles, such as technical difficulties and usability challenges, highlighting the necessity for comprehensive solutions.
- The research delineates accessibility obstacles faced by elderly individuals and marginalized populations, recommending targeted design enhancements to mitigate the digital gap.
- It offers region-specific data for Saudi Arabia, linking enhancements in mobile health applications with national healthcare objectives under Vision 2030.

## 2. Methods

### 2.1. Study Design

This research utilized a cross-sectional design focusing on patients who have utilized the Sehaty app in Saudi Arabia. The cross-sectional approach facilitated a temporal assessment of the barriers encountered by app users at a designated moment.

### 2.2. Population and Sampling

The study focused on patients with chronic diseases who frequently use the Sehaty app in Saudi Arabia. To ensure the inclusion of individuals actively engaged in managing their health through the app, a purposive sampling method was employed. This approach allowed the study to gather insights from participants with firsthand experience using Sehaty for chronic disease management. Eligibility criteria required participants to have been diagnosed with at least one chronic disease, such as diabetes, hypertension, or cardiovascular conditions, and to demonstrate consistent engagement with the app for accessing healthcare services, scheduling appointments, or monitoring their health. Patients with infrequent app usage or limited digital health literacy were excluded to maintain a focus on those who rely on Sehaty as a primary health resource. In total, 344 responses were collected, serving as the foundation for subsequent data analysis.

### 2.3. Data Collection Instrument

This study employed a structured questionnaire to assess the barriers to the utilization of the Sehaty mobile health application. The questionnaire was developed based on an extensive review of the literature, identifying 10 primary variables recognized as key challenges in mHealth adoption. These variables included Technical Barriers (e.g., system crashes, slow responsiveness, and frequent bugs), Usability Barriers (e.g., ease of navigation, task completion time, and interface complexity), Support and Training Barriers (e.g., availability of user support and clarity of instructions), Accessibility Barriers (e.g., usability for individuals with disabilities and readability of text), and Privacy and Security Barriers (e.g., concerns about data security and trust in information handling). Other key variables assessed were Communication and Interaction Barriers (e.g., ability to contact healthcare providers and responsiveness of messaging features), Functionality Barriers (e.g., availability of essential features and accuracy of medical data), User Satisfaction Barriers (e.g., overall confidence in using the app and perceived usefulness), Cost and Accessibility Barriers (e.g., internet access, device compatibility, and app memory requirements), and Time and Productivity Barriers (e.g., efficiency of app tasks and additional steps required for healthcare management).

Each of these variables was operationalized through 4 to 5 items, resulting in a total of 45 structured items in the questionnaire. To supplement the quantitative data, an open-ended question was included to allow participants to elaborate on specific barriers they encountered while using the Sehaty app. This qualitative component provided contextual depth, helping to identify emerging user concerns that may not have been fully captured by the structured survey items.

The questionnaire was divided into four main sections to ensure clarity and comprehensiveness. The first section included an assurance letter outlining the purpose and scope of the study. Participants were informed that their participation was voluntary and that the estimated time required to complete the questionnaire was 10–15 min. The letter also assured respondents of data confidentiality and security, emphasizing that all responses would be anonymized and used exclusively for research purposes.

The second section focused on demographic information, capturing key characteristics, such as age, gender, level of education, frequency of Sehaty app usage, and digital health literacy levels. This section allowed for the analysis of potential variations in usability perceptions across different user groups. The third section comprised 45 Likert scale items, measuring the 10 usability and barrier-related variables. Participants were asked to rate their level of agreement with each statement on a 5-point Likert scale (1 = Strongly Disagree, 5 = Strongly Agree). This section provided quantitative insights into the specific usability challenges that influenced the adoption and effectiveness of the Sehaty application. The fourth and final section featured an open-ended question, allowing participants to describe additional usability barriers or challenges they faced while using Sehaty. This qualitative component enriched the dataset by capturing user experiences and concerns that may not have been fully reflected in the structured items.

#### Pilot Study

Before launching the full-scale data collection, the questionnaire underwent a pilot study to test its reliability, validity, and clarity. A total of 13 participants, representative of the target population, were recruited to complete the questionnaire and provide feedback on its clarity, comprehensiveness, and ease of understanding.

Several key aspects were evaluated during the pilot study. Item clarity and wording were assessed to ensure that all questions were clearly phrased and easily understood. The relevance of the questions was reviewed to confirm that the questionnaire effectively captured the intended barriers to Sehaty app usage. Additionally, the time required for completion was measured to prevent respondent fatigue while maintaining a comprehensive assessment.

To ensure internal consistency and reliability, Cronbach’s alpha was calculated for each of the 10 variables. All variables demonstrated acceptable reliability levels (α > 0.70), confirming the internal coherence of the scale. Furthermore, the questionnaire underwent face and content validity assessments by experts in digital health and usability research, who ensured the instrument adequately covered all relevant usability dimensions. Based on the feedback received, minor modifications were made to improve question clarity and optimize item wording before the full-scale implementation.

### 2.4. Data Collection and Analysis Procedure

Data analysis was conducted using SPSS v29 and R software (Version 4.3.0) to ensure a comprehensive examination of the study’s findings. Descriptive statistics, including frequencies, percentages, and means, were computed for each of the 45 items to summarize participants’ responses. This analysis provided insights into the prevalence and intensity of perceived barriers to the utilization of mobile health applications.

To assess the reliability of the questionnaire, Cronbach’s alpha was calculated for both the entire instrument and the 10 key variables, each representing distinct dimensions of barriers to Sehaty app utilization. The results confirmed that the questionnaire demonstrated strong internal consistency, making it suitable for further statistical analysis. Additionally, inferential statistics were applied to examine statistically significant differences in participants’ responses across various demographic groups. Correlation analyses were conducted to explore relationships among the main variables, offering deeper insights into the interconnected nature of barriers to mobile health application utilization.

R software (Version 4.3.0) was employed for data visualization, particularly in representing correlations among the 10 primary variables (themes). Graphical representations provided a clearer understanding of the relationships between usability, accessibility, privacy, technical challenges, and other barriers, highlighting potential areas requiring further intervention. In addition to quantitative analysis, qualitative responses from open-ended questions were analyzed using a thematic approach. Responses were coded and categorized into distinct themes, with frequencies calculated to determine the most commonly reported barriers. This thematic analysis provided valuable contextual insights, complementing the quantitative findings and identifying specific user concerns that may not have been captured through structured survey items.

To ensure the validity and reliability of the findings, collinearity diagnostics were also conducted. Variance inflation factor (VIF) and Tolerance values were computed to assess potential collinearity among independent variables. The results indicated that all VIF values were below 10 and Tolerance values exceeded 0.1, confirming that collinearity was not a significant concern. Therefore, all variables were retained in the analysis without modification.

## 3. Results

As seen in Table 1, the questionnaire’s reliability was evaluated using Cronbach’s alpha, demonstrating robust internal consistency among all variables and the overall instrument. The Cronbach’s alpha values for individual variables varied from 0.76 (Functionality Barriers) to 0.92 (Accessibility Barriers), demonstrating strong reliability for each item subset. The questionnaire exhibited exceptional reliability, evidenced by a Cronbach’s alpha of 0.95, affirming that the tool is highly dependable for assessing barriers to the use of the Sehaty app. The results indicate that the questionnaire is reliable and effectively captures participants’ perceptions across several aspects.

**Figure 1:**
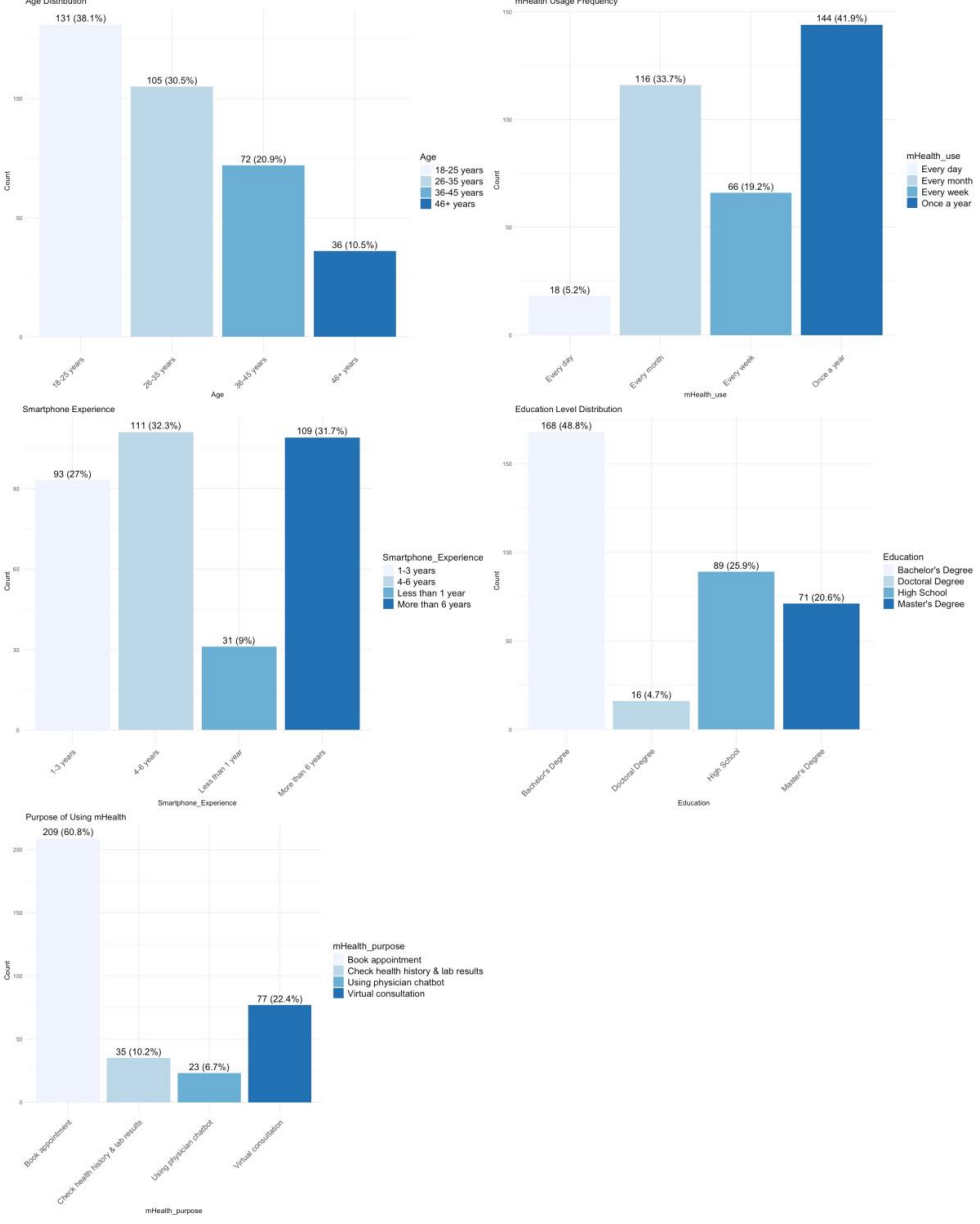
Patient demographic distribution by app usage, smartphone experience, age, education, and purpose of mHealth use.

Figure 1 presents the demographic distribution of participants categorized by app usage frequency, smartphone experience, age, education level, and purpose of using mHealth applications, illustrating key trends in mHealth adoption across different user groups. The data indicate that younger adults, particularly those aged 26–35 years (105 participants, 25.1%), represent the largest group of mHealth users, followed by those in the 18–25 years category (79 participants, 20.2%). The frequency of app usage also varies, with a significant proportion of users engaging with the Sehaty app daily or weekly (144 users, 34.1%), reflecting a high reliance on digital healthcare services. Similarly, smartphone experience data reveal that most participants have been using smartphones for over 6 years (190 participants, 71%), demonstrating a well-established familiarity with mobile technology. In contrast, only 31 participants (11%) reported having 1 to 3 years of smartphone experience, suggesting that digital literacy is generally high within this sample.

Education level further influences mHealth adoption, as the majority of users hold a bachelor’s degree (82 participants, 29.8%), followed by those with a master’s degree (78 participants, 28.3%), while a smaller segment of respondents have only a high school diploma (54 participants, 14.7%). This trend aligns with previous research, suggesting that higher education levels correspond with greater engagement in digital health solutions. The purpose of using mHealth applications varies, with the most common reason being appointment booking (79 users, 39.5%), followed by accessing test results (29 users, 29.5%), while fewer participants used it for teleconsultation or health record management. These findings highlight the increasing role of mHealth applications in enhancing healthcare accessibility and efficiency. They also emphasize the need for user-friendly interfaces and improved usability features, ensuring that digital healthcare tools remain accessible and efficient for diverse user demographics, particularly those with lower digital literacy levels.

**Figure 2:**
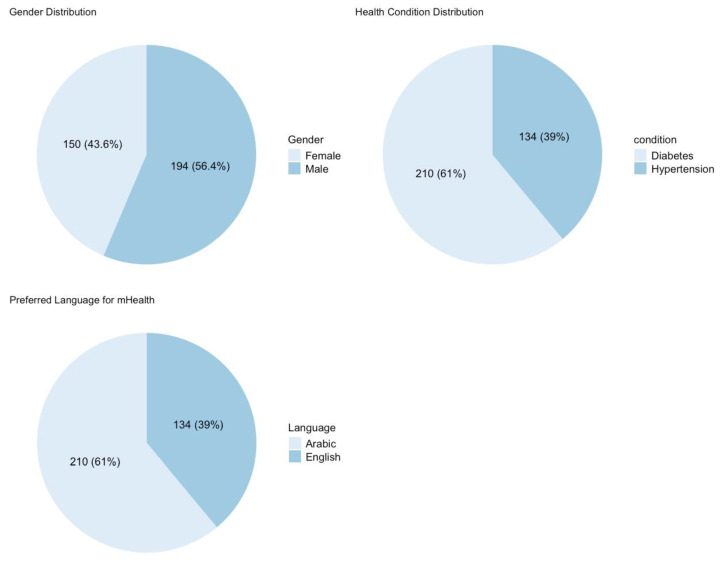
Patient demographic distribution by gender, condition, and the app language used.

Figure 2 illustrates the demographic distribution of participants categorized by gender, health condition, and preferred language for mHealth applications, providing insights into the user characteristics and language preferences that may influence mHealth adoption and engagement. The first chart presents the gender distribution, revealing that the majority of participants were male (194 participants, 56.4%), while females constituted 150 participants (43.6%). This suggests a relatively balanced gender representation among the respondents, though with a slightly higher proportion of male users. The second chart illustrates the distribution of health conditions among participants, with hypertension being the most commonly reported condition (210 participants, 61%), followed by diabetes (134 participants, 39%). These findings indicate that a significant portion of Sehaty users rely on the application for managing chronic diseases, reinforcing the importance of mHealth platforms in supporting long-term disease management.

The third chart highlights the preferred language for mHealth applications, demonstrating a strong preference for Arabic (210 participants, 61%), compared to English (134 participants, 39%). This emphasizes the need for mHealth platforms to prioritize Arabic language support, ensuring that content, navigation, and user assistance are fully accessible to Arabic-speaking users. The overall findings suggest that gender, health conditions, and language preferences play a crucial role in shaping mHealth usability and adoption. Ensuring that digital health services cater to linguistic diversity and chronic disease management needs could enhance engagement and effectiveness for a broader user base.

Table 2 presents key findings on the challenges users face in utilizing the Sehaty application, with a particular focus on technical and usability issues. The most significant technical barrier identified was frequent crashes and application instability, which received the highest mean score (mean = 4.02). Users also reported difficulties in locating information within the application (mean = 3.84), indicating navigation challenges, while layout design was comparatively less criticized (mean = 2.09).

Issues related to support, accessibility, and privacy were also notable. Many participants highlighted a lack of instructional support or training for application use (mean = 2.22), while accessibility concerns, particularly among older users, emerged as a significant barrier (mean = 3.79). Additionally, privacy concerns regarding data security and transparency were prominent, with the statement “The application does not provide enough information about how my data is used” receiving a mean score of 3.93.

Challenges in communication and interaction further impacted the user experience. Messaging and chat features were particularly problematic (mean = 4.01), while functionality and user satisfaction barriers pointed to key areas needing improvement. Users reported difficulties in tracking health data effectively (mean = 4.02) and expressed low confidence in using the application (mean = 3.96). Although cost concerns were minimal (mean = 2.01), consistent internet access was highlighted as a significant obstacle (mean = 3.99). Overall, these findings underscore the need for enhanced usability, better support and training, improved accessibility features, and greater transparency in data security to comprehensively address these barriers.

**Figure 3:**
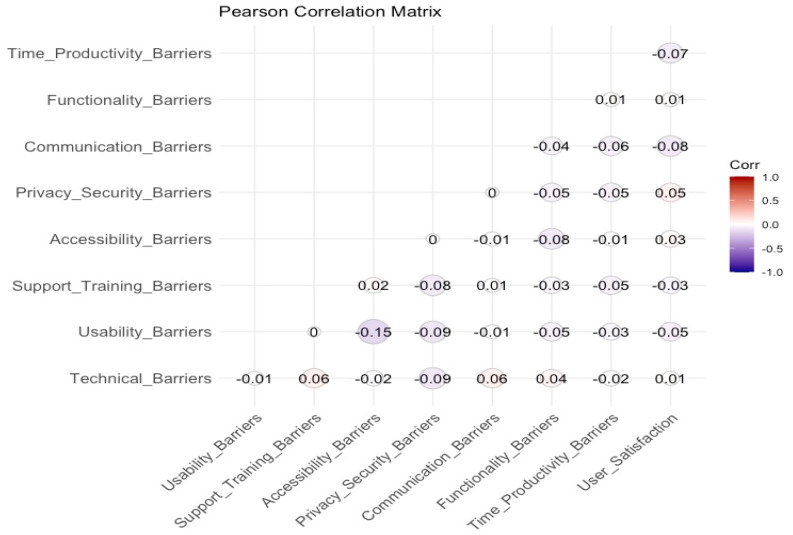
Correlation between main variables.

The correlation analysis, as illustrated in Figure 3, reveals moderate correlations between accessibility and support barriers, as well as between time and productivity constraints and cost-related barriers. These findings suggest that accessibility challenges often stem from insufficient training or support, while time management inefficiencies are closely linked to broader accessibility limitations. In contrast, weak correlations between factors such as cost and technical barriers indicate that certain challenges may operate independently. These results underscore the need for a comprehensive approach to application enhancement, with a focus on technical reliability, usability, security, and accessibility to improve user satisfaction and effectively mitigate barriers.

Further examination of the correlation analysis highlights significant relationships among the primary barriers faced by users. Notably, strong correlations were observed among technical, usability, and functionality barriers, indicating that technical issues, such as crashes and system instability, have a direct negative impact on the user experience and essential application features. Additionally, user satisfaction was significantly associated with privacy and security concerns, emphasizing the crucial role of data protection and transparency in fostering user trust and confidence.

These findings reinforce the interconnected nature of user challenges, suggesting that addressing one issue, such as enhancing technical stability or improving accessibility, can lead to broader improvements across multiple aspects of the user experience.

**Figure 4:**
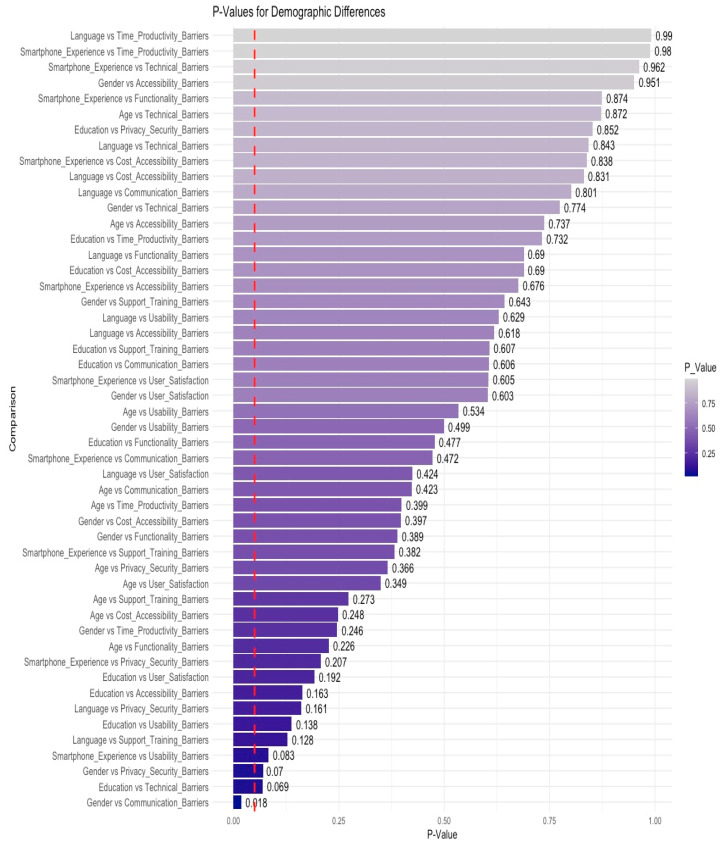
*p*-values for demographic differences.

As illustrated in Figure 4, the analysis of statistical differences across demographic factors and perceived barriers reveals that most comparisons do not exhibit statistically significant variation. The majority of p-values exceed the conventional threshold of 0.05, indicating that demographic characteristics, such as gender, age, education, language, and smartphone experience, do not significantly influence participants’ perceptions of the identified barriers. However, a statistically significant difference was observed in the relationship between gender and communication barriers (*p* = 0.018), suggesting that males and females experience communication-related challenges in distinct ways.

Additionally, while not reaching statistical significance, the relationships between education and technical barriers (*p* = 0.069) and smartphone experience and usability barriers (*p* = 0.083) approached significance, indicating potential trends that warrant further investigation. In contrast, comparisons such as language and time productivity barriers (*p* = 0.991) and smartphone experience and time productivity barriers (*p* = 0.989) yielded high *p*-values, suggesting that perceptions of time and productivity barriers remain consistent across participant groups.

The limited number of statistically significant differences suggests that the potential bias introduced by convenience sampling is minimal. If significant bias were present, greater disparities among demographic subgroups would be expected. The consistency of responses across various categories strengthens the study’s validity, indicating that demographic differences do not substantially influence the overall findings. However, the notable disparity in gender-based communication barriers highlights the need for further exploration, potentially through qualitative studies or larger, more diverse sample sizes, to better understand gender-specific communication challenges in mHealth applications.

**Figure 5:**
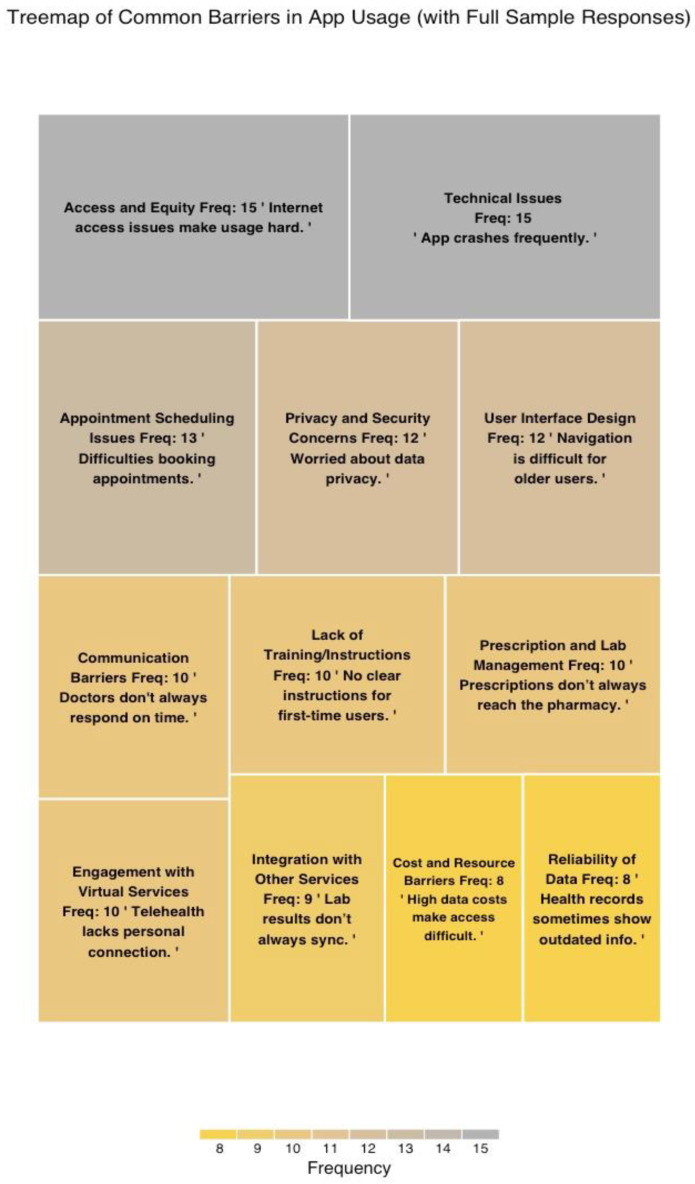
Responses to open-ended question: what other barriers you face when using Sehatty app.

The qualitative analysis of open-ended responses identified 12 key themes related to the challenges patients face when using mHealth applications, including telehealth, virtual consultations, and follow-up services (see Figure 5). The most frequently mentioned themes were Technical Issues and Access and Equity, each cited in 15 responses (12.1%). Patients frequently expressed dissatisfaction with recurrent application failures, prolonged loading times, and unreliable internet connectivity. Similarly, concerns regarding equitable access, including unstable internet connections and language barriers, were prominent, emphasizing the need for improved infrastructure and inclusive application design.

Other notable themes included Privacy and Security Concerns (12 responses, 9.7%) and Appointment Scheduling Issues (13 responses, 10.5%). Participants raised concerns about the storage and protection of their medical data, as well as difficulties in securing available appointment slots and obtaining timely booking confirmations. Additionally, themes such as User Interface Design (12 responses, 9.7%) and Engagement with Virtual Services (10 responses, 8.1%) highlighted challenges in navigating the application and a reluctance to fully adopt virtual consultations due to a perceived lack of human interaction.

Less frequently mentioned but still significant themes included Cost and Resource Barriers (8 responses, 6.5%) and Reliability of Data (8 responses, 6.5%), reflecting concerns about affordability and occasional inaccuracies in health records. These findings provide valuable insights into the barriers that patients face, underscoring opportunities for enhancing application design, functionality, and accessibility to improve the overall mHealth experience.

## 4. Discussion

The findings of this study reveal significant barriers to the utilization of the Sehaty app among patients with chronic diseases in Saudi Arabia, with technical and usability issues emerging as the most critical challenges. Frequent app crashes, slow responsiveness, and navigation difficulties were identified as primary obstacles, contributing to user dissatisfaction and reduced engagement. These findings align with the work of Giebel, Abels [9], who identified technical instability as a major deterrent to digital health adoption, negatively impacting user trust and engagement. Similarly, usability concerns, particularly difficulties in locating essential features and the complexity of the user interface, support the observations of Zhou, Bao [8], who emphasized the role of poor design and navigation challenges in limiting mHealth adoption. Comparable results were reported by Nurgalieva, O’Callaghan [25], who highlighted security and privacy concerns as additional obstacles in mHealth use, leading to diminished patient confidence.

The findings of this study align with prior research on the usability of the Sehaty application while also revealing important differences in user experiences. Similar to Ali [26], the results indicate that although many users found Sehaty easy to use, a significant portion remained neutral, suggesting that usability challenges persist, particularly in terms of navigation and accessibility. Banwas, Ajina [27] further reinforce this observation, highlighting that urban users reported higher ease of use and satisfaction, whereas provincial users faced greater challenges, likely due to differences in digital literacy and access to high-speed internet. Additionally, Dawood and Alkadi [17] provide a comparative assessment of Sehhaty’s usability and reliability, demonstrating that while the application performed moderately well, users remained skeptical about its ability to fully replace traditional in-person healthcare services. This finding is consistent with the usability barriers identified in the present study, particularly regarding technical instability, slow responsiveness, and navigation difficulties, which may undermine user confidence in mHealth solutions. While Ali [26] noted that Tawakkalna was perceived as more user-friendly than Sehaty, the present study, along with Banwas, Ajina [27], emphasizes that Sehaty’s usability varies across different user demographics, with urban users reporting a more favorable experience compared to those in underserved areas.

A similar trend is observed in European mHealth research, where studies have noted that a lack of user-friendly technology and simple user interfaces remains a major barrier to adoption. For example, Stefanicka-Wojtas and Kurpas [28] identified that even in well-developed healthcare ecosystems, digital health platforms often struggle with accessibility issues, leading to disparities in user experiences across different demographic groups. Similarly, Hassanaly and Dufour [23] found that mHealth applications in Europe suffer from usability challenges related to interface complexity and inconsistent user experiences, particularly for older adults and individuals with limited digital proficiency. Collectively, these findings highlight the need for targeted usability improvements, including enhanced interface design, system stability, and accessibility features, to ensure that Sehaty effectively meets the needs of diverse user populations.

Beyond technical challenges, privacy and security concerns emerged as significant barriers, as participants expressed apprehension regarding data safety and transparency. This is consistent with findings from Kansiime, Atusingwize [1], who highlighted privacy concerns as a major impediment to mHealth adoption, particularly when users lack clarity about data collection and usage policies. A systematic review by Alhammad, Alajlani [29] further corroborates these concerns, emphasizing that data confidentiality issues significantly impact user trust and willingness to engage with mHealth solutions. Another systematic review conducted in the United States identified legal challenges, particularly privacy regulations, as a significant barrier to mHealth adoption. The study emphasized that stringent compliance requirements not only complicate implementation but also hinder the scalability and widespread integration of digital healthcare solutions [23]. Moreover, a study conducted in Europe by Iwaya, Ahmad [30] highlighted that inadequately implemented security protocols not only jeopardize patient data but also erode trust in digital healthcare applications.

Accessibility barriers were also evident, particularly among older adults and individuals with disabilities, reflecting challenges in interface design, navigation, and ease of use. These findings are consistent with Byambasuren, Byambasuren, Beller [10], who noted that underserved populations, including the elderly, often struggle with mHealth adoption due to insufficient accessibility features and limited technical support. The study by Liu, Lu [31] supports this, suggesting that self-efficacy and privacy concerns influence digital health adoption, particularly for users with lower digital literacy. Enhancing accessibility through larger fonts, simplified navigation, voice assistance, and multilingual support could significantly improve usability for diverse user groups.

Interestingly, cost was not identified as a major barrier, contrasting with findings from studies conducted in low-income settings, where financial constraints hinder mHealth adoption. This discrepancy may be attributed to the subsidized nature of the Sehaty app and its alignment with Saudi Arabia’s Vision 2030 initiative, which aims to expand digital healthcare access. However, time-related barriers, such as inefficiencies in task completion, redundant workflows, and slow processing speeds, were frequently reported, echoing the concerns raised by [11], who found that poorly optimized mHealth applications fail to streamline healthcare management tasks. This finding aligns with the study by Alenoghena, Onumanyi [32], which discusses how technological limitations, including weak internet infrastructure, can hinder the efficiency of digital health solutions.

These findings underscore the interconnected nature of user challenges, where improvements in technical stability and usability could also enhance time efficiency and overall user satisfaction. Additionally, further research could explore the intersection of security measures, usability enhancements, and accessibility improvements to create a more comprehensive approach to overcoming mHealth adoption barriers. With the global expansion of digital health solutions, addressing and mitigating barriers to mHealth adoption is critical to ensuring their effective integration into healthcare systems. As governments and healthcare providers increasingly incorporate digital health technologies, the findings of this study provide valuable insights for the development of user-centered, secure, and accessible mHealth platforms. In the post-pandemic era, where reliance on telehealth services has significantly increased, overcoming usability and security challenges has become more essential than ever. By informing future policy and design improvements, this research can contribute to advancing digital health equity, ensuring that mHealth solutions effectively serve diverse populations and enhance healthcare delivery at scale.

To enhance the practical applicability of the proposed improvements, a structured approach is recommended, categorizing them into short-term, mid-term, and long-term strategies to ensure systematic and sustainable enhancements. Short-term strategies should prioritize addressing critical technical issues, including reducing application crashes, improving system responsiveness, and optimizing navigation through immediate software updates and user interface refinements. Mid-term strategies should focus on enhancing data privacy measures, expanding accessibility features for individuals with disabilities, and implementing comprehensive user training programs to improve digital literacy and engagement. Long-term strategies should involve leveraging advanced analytics to monitor user behavior, integrating AI-driven personalization to enhance usability, and conducting regular user experience assessments to facilitate continuous improvements. By adopting a phased and strategic approach, the Sehaty app can achieve progressive enhancements, ultimately leading to higher user adoption, improved engagement, and long-term satisfaction in the evolving digital healthcare landscape.

### 4.1. Strengths and Limitations

This study offers several strengths. It provides a comprehensive evaluation of barriers to mHealth application adoption, focusing on the Sehaty app, a widely used digital health platform in Saudi Arabia. By examining technical, usability, privacy, accessibility, and time-related constraints, the study presents a holistic perspective on user challenges. Additionally, the inclusion of diverse demographic groups, including older adults and individuals with disabilities, enhances the study’s applicability to marginalized populations. The use of a systematic questionnaire ensures consistency across participants, while the application of statistical analysis, including correlation assessments, adds depth to the findings.

Nonetheless, some limitations should be acknowledged. The study’s reliance on self-reported data may introduce response bias, as participants’ subjective experiences may not fully reflect objective assessments of app performance. Furthermore, the cross-sectional design limits the ability to establish causal relationships between perceived barriers and app adoption patterns. While the sample size is substantial, it may not fully represent all patient demographics, particularly those in rural or underserved regions. Additionally, the findings are specific to the Sehaty app and may not be generalizable to other mHealth platforms in different cultural or healthcare contexts.

### 4.2. Future Research Directions

Future research could address these limitations by adopting longitudinal study designs to assess changes in user experiences over time. Incorporating objective performance metrics, such as application log data and error reports, could complement self-reported insights and provide a more accurate representation of usability challenges. Furthermore, comparative analyses across various mHealth platforms could offer broader insights into best practices for enhancing digital health adoption. Given the gender-based differences observed in communication barriers, future studies could also explore these disparities through qualitative methods or larger, more diverse samples to better understand how communication preferences and challenges vary across user groups.

## 5. Conclusions

The findings of this study emphasize the critical need for targeted improvements in technical stability, usability, security, and accessibility to enhance the adoption and effectiveness of the Sehaty app. While technical and usability issues emerged as the most prominent barriers, privacy concerns, accessibility limitations, and time-related inefficiencies also significantly affected user experiences. The study highlights the interconnected nature of these challenges, suggesting that enhancing key aspects of the application, such as stability, navigation, and security, could have positive ripple effects on user confidence, engagement, and overall satisfaction.

To address these challenges, the study proposes actionable recommendations for improving accessibility and security, including integrating assistive technologies for users with disabilities, enhancing data transparency and security protocols, and streamlining navigation and task completion workflows. Additionally, future research should incorporate longitudinal studies to assess the long-term impact of these improvements on user adoption and satisfaction. A user-centered design approach, incorporating accessibility enhancements, robust security measures, and optimized workflows, is essential for maximizing the impact of digital health initiatives and ensuring equitable healthcare access in Saudi Arabia.

## Figures and Tables

**Table 1:** Scale reliability using Cronbach’s alpha test.

Variables	Number of Items	Cronbach’s Alpha
Technical Barriers	5	0.85
Usability Barriers	5	0.88
Accessibility Barriers	4	0.92
Support and Training Barriers	5	0.85
Privacy and Security Barriers	4	0.89
Communication and Interaction Barriers	4	0.90
Functionality Barriers	4	0.76
User Satisfaction Barriers	5	0.83
Cost and Accessibility Barriers	5	0.83
Time and Productivity Barriers	4	0.89
Entire questionnaire	45	0.95

**Table 2:** Barriers distribution with percentages and mean.

Items	Strongly Disagree	Disagree	Neutral	Agree	Strongly Agree	Mean
Technical Barriers						
The application often crashes or stops working while I am using it.	27 (7.85%)	26 (7.56%)	21 (6.1%)	113 (32.85%)	157 (45.64%)	4.02
The application is slow and unresponsive.	107 (31.1%)	136 (39.53%)	17 (4.94%)	48 (13.95%)	36 (10.47%)	2.32
The application often fails to connect to the internet.	141 (40.99%)	135 (39.24%)	21 (6.1%)	23 (6.69%)	24 (6.98%)	2.02
I experience frequent bugs or errors while using the application.	99 (28.78%)	135 (39.24%)	33 (9.59%)	45 (13.08%)	32 (9.3%)	2.37
The application doesn’t work well on my device (phone, tablet, etc.).	117 (34.01%)	138 (40.12%)	22 (6.4%)	30 (8.72%)	37 (10.76%)	2.22
Usability Barriers						
The application is difficult to use without help.	95 (27.62%)	120 (34.88%)	40 (11.63%)	52 (15.12%)	37 (10.76%)	2.4
I find it hard to navigate through the different features of the application.	105 (30.52%)	114 (33.14%)	42 (12.21%)	50 (14.53%)	33 (9.59%)	2.29
It takes too long to complete tasks, like booking appointments or viewing records, in the application.	110 (31.98%)	122 (35.47%)	35 (10.17%)	45 (13.08%)	32 (9.3%)	2.36
The application’s layout is confusing and not user-friendly.	124 (36.05%)	132 (38.37%)	31 (9.01%)	38 (11.05%)	19 (5.52%)	2.09
I often struggle to find the information I need within the application.	25 (7.27%)	68 (19.77%)	20 (5.81%)	110 (31.98%)	121 (35.17%)	3.84
Support and Training Barriers						
I did not receive any instructions or training on how to use the application.	95 (27.62%)	110 (31.98%)	48 (13.95%)	45 (13.08%)	46 (13.37%)	2.22
There is no help or support available when I have issues with the application.	100 (29.07%)	103 (29.94%)	50 (14.53%)	42 (12.21%)	49 (14.24%)	2.17
The instructions provided in the application are not clear or helpful.	94 (27.33%)	111 (32.27%)	49 (14.24%)	50 (14.53%)	40 (11.63%)	2.24
I do not know how to get assistance if the application doesn’t work.	101 (29.36%)	109 (31.69%)	41 (11.92%)	48 (13.95%)	45 (13.08%)	2.22
Accessibility Barriers						
The application is not accessible for people with disabilities (e.g., visual, hearing impairments).	105 (30.52%)	118 (34.3%)	27 (7.85%)	50 (14.53%)	44 (12.79%)	2.17
The font size and layout of the application make it difficult to read or use.	28 (8.14%)	55 (15.99%)	29 (8.43%)	140 (40.7%)	92 (26.74%)	3.88
The application is difficult to use for older people.	32 (9.3%)	58 (16.86%)	30 (8.72%)	130 (37.79%)	94 (27.33%)	3.79
I find it difficult to input information into the application.	110 (31.98%)	112 (32.56%)	45 (13.08%)	47 (13.66%)	30 (8.72%)	2.37
Privacy and Security Barriers						
I am concerned about the privacy of my personal and medical information in the application.	40 (11.63%)	39 (11.34%)	36 (10.47%)	99 (28.78%)	130 (37.79%)	3.9
I am worried that unauthorized people could access my data through the application.	98 (28.49%)	108 (31.4%)	37 (10.76%)	43 (12.5%)	58 (16.86%)	2.44
The application does not provide enough information about how my data is used.	38 (11.05%)	40 (11.63%)	40 (11.63%)	92 (26.74%)	134 (38.95%)	3.93
I do not trust the security of the application when it comes to protecting my medical records.	34 (12.01%)	37 (13.07%)	35 (12.37%)	128 (45.23%)	49 (17.31%)	3.93
Communication and Interaction Barriers						
The application makes it difficult to communicate with my healthcare providers.	125 (36.34%)	110 (31.98%)	55 (15.99%)	35 (10.17%)	19 (5.52%)	2.1
I do not receive timely responses from my healthcare provider through the application.	96 (27.91%)	99 (28.78%)	48 (13.95%)	64 (18.6%)	37 (10.76%)	2.41
I find it challenging to use the messaging or chat features in the application.	32 (9.22%)	33 (9.51%)	40 (11.53%)	110 (31.7%)	132 (38.04%)	4.01
The application does not allow me to interact effectively with my doctor or healthcare team.	121 (35.17%)	108 (31.4%)	52 (15.12%)	35 (10.17%)	28 (8.14%)	2.26
Functionality Barriers						
The application lacks important features I need (e.g., scheduling appointments, viewing prescriptions).	105 (30.52%)	123 (35.76%)	45 (13.08%)	45 (13.08%)	26 (7.56%)	2.25
The application does not provide updated or accurate medical information.	110 (31.98%)	113 (32.85%)	39 (11.34%)	40 (11.63%)	42 (12.21%)	2.29
I find it hard to track my health or medical data using the application.	35 (11.67%)	48 (16%)	42 (14%)	112 (37.33%)	63 (21%)	4.02
The application doesn’t integrate well with other health services I use.	125 (33.69%)	140 (37.74%)	48 (12.94%)	28 (7.55%)	30 (8.09%)	2.2
User Satisfaction Barriers						
I do not feel confident using the application.	21 (6.48%)	50 (15.43%)	24 (7.41%)	115 (35.49%)	114 (35.19%)	3.96
I don’t find the application helpful for managing my health.	22 (6.79%)	46 (14.2%)	25 (7.72%)	116 (35.8%)	115 (35.49%)	4.01
The application is not improving my experience with healthcare.	130 (37.79%)	135 (39.24%)	42 (12.21%)	26 (7.56%)	11 (3.2%)	2.18
I would prefer to manage my healthcare without using this application.	31 (10.2%)	32 (10.53%)	26 (8.55%)	108 (35.53%)	107 (35.2%)	3.94
I would not recommend this application to others.	112 (32.56%)	117 (34.01%)	41 (11.92%)	64 (18.6%)	10 (2.91%)	2.29
Cost and Accessibility Barriers						
The application is too expensive or requires paid features to access important services.	97 (28.2%)	102 (29.65%)	38 (11.05%)	55 (15.99%)	52 (15.12%)	2.01
I cannot use the application because I don’t have consistent access to the internet.	102 (29.65%)	104 (30.23%)	35 (10.17%)	48 (13.95%)	55 (15.99%)	3.99
My device (phone, tablet) is too old or incompatible with the application.	98 (28.49%)	97 (28.2%)	39 (11.34%)	50 (14.53%)	60 (17.44%)	2.33
The application requires too much data or memory on my device.	99 (28.78%)	98 (28.49%)	35 (10.17%)	52 (15.12%)	60 (17.44%)	3.83
Time and Productivity Barriers						
It takes too long to accomplish tasks in the application compared to other methods.	110 (31.98%)	106 (30.81%)	40 (11.63%)	38 (11.05%)	50 (14.53%)	3.89
The application adds unnecessary steps to managing my healthcare.	104 (30.23%)	103 (29.94%)	38 (11.05%)	39 (11.34%)	60 (17.44%)	2.29
Using the application does not save me time when managing my health.	108 (31.4%)	112 (32.56%)	42 (12.21%)	42 (12.21%)	40 (11.63%)	3.91
The application slows down my ability to book appointments or access services.	107 (31.1%)	105 (30.52%)	39 (11.34%)	50 (14.53%)	43 (12.5%)	3.84

## Data Availability

The datasets used and analyzed during the current study are available from the corresponding author on reasonable request.
